# Cost–effectiveness of a comprehensive programme for drug-resistant tuberculosis in China

**DOI:** 10.2471/BLT.14.146274

**Published:** 2015-09-14

**Authors:** Christopher Fitzpatrick, Zhang Hui, Wang Lixia, Li Renzhong, Ruan Yunzhou, Chen Mingting, Zhao Yanlin, Zhao Jin, Su Wei, Xu Caihong, Chen Cheng, Timothy Alston, Qu Yan, Lv Chengfei, Fu Yunting, Huan Shitong, Sun Qiang, Fabio Scano, Daniel P Chin, Katherine Floyd

**Affiliations:** a World Health Organization, Geneva, Switzerland.; b National Center for Tuberculosis Control and Prevention, Chinese Center for Disease Control, Beijing, China.; c Center for Health Management and Policy, Key Lab of Health Economic and Policy Research of Ministry of Health, Shandong University, No 44 Wenhua Rd, Mailbox 128, Jinan, Shandong 250012, China.; d Bill & Melinda Gates Foundation, Beijing, China.; e World Health Organization, Beijing, China.

## Abstract

**Objective:**

To investigate the cost–effectiveness of a comprehensive programme for drug-resistant tuberculosis launched in four sites in China in 2011.

**Methods:**

In 2011–2012, we reviewed the records of 172 patients with drug-resistant tuberculosis who enrolled in the comprehensive programme and we collected relevant administrative data from hospitals and China’s public health agency. For comparison, we examined a cohort of 81 patients who were treated for drug-resistant tuberculosis in 2006−2009. We performed a cost–effectiveness analysis, from a societal perspective, that included probabilistic uncertainty. We measured early treatment outcomes based on three-month culture results and modelled longer-term outcomes to facilitate estimation of the comprehensive programme’s cost per disability-adjusted life-year (DALY) averted.

**Findings:**

The comprehensive programme cost 8837 United States dollars (US$) per patient treated. Low enrolment rates meant that some fixed costs were higher, per patient, than expected. Although the comprehensive programme appeared 30 times more costly than the previous one, it resulted in greater health benefits. The comprehensive programme, which cost US$ 639 (95% credible interval: 112 to 1322) per DALY averted, satisfied the World Health Organization’s criterion for a very cost–effective intervention.

**Conclusion:**

The comprehensive programme, which included rapid screening, standardized care and financial protection, improved individual outcomes for MDR tuberculosis in a cost-effective manner. To support post-2015 global heath targets, the comprehensive programme should be expanded to non-residents and other areas of China.

## Résumé

**Objectif:**

Étudier le rapport coût-efficacité d'un programme global de lutte contre la tuberculose pharmacorésistante lancé en 2011 dans quatre régions chinoises.

**Méthodes:**

En 2011-2012, nous avons analysé les dossiers de 172 patients atteints de tuberculose pharmacorésistante qui s'étaient inscrits à ce programme global et nous avons collecté des données administratives pertinentes auprès de différents hôpitaux et de l'agence de santé publique chinoise. À titre de comparaison, nous avons examiné une cohorte de 81 patients soignés pour une tuberculose pharmacorésistante entre 2006 et 2009. Nous avons réalisé une analyse coût-efficacité, sur le plan sociétal, qui incluait une incertitude probabiliste. Nous avons mesuré les résultats du traitement précoce en fonction de résultats de culture sur trois mois et modélisé les résultats à long terme pour faciliter l'estimation du coût du programme global par année de vie corrigée du facteur incapacité (AVCI) évitée.

**Résultats:**

Le programme global coûte 8837 dollars des États-Unis (US$) par patient soigné. Le faible taux d'inscription signifiait que certains coûts fixes par patient étaient plus importants que prévu. Bien que ce programme global se soit révélé 30 fois plus cher que le précédent, il a débouché sur des avantages accrus en matière de santé. Le programme global, qui coûte 639 US$ (intervalle de crédibilité à 95 %: 112 à 1322) par AVCI évitée, satisfait au critère de l'Organisation mondiale de la Santé définissant une intervention très rentable.

**Conclusion:**

Le programme global, qui incluait un dépistage rapide, des soins standardisés et une protection financière, a amélioré les résultats des patients atteints de tuberculose multirésistante en limitant les dépenses. Afin de soutenir les objectifs mondiaux pour l'après-2015 en matière de santé, le programme global devrait être élargi aux non-résidents et à d'autres régions de Chine.

## Resumen

**Objetivo:**

Investigar la costoeficacia de un programa integral para la tuberculosis farmacorresistente lanzado en cuatro localizaciones de China en 2011.

**Métodos:**

En 2011–2012, se revisaron los informes de 172 pacientes con tuberculosis farmacorresistente que se habían inscrito en el programa integral y se recogieron datos administrativos relevantes de hospitales y la agencia de salud pública de China. Como término de comparación, se examinó un cohorte de 81 pacientes que habían sido tratados para la tuberculosis farmacorresistente en 2006−2009. Se llevó a cabo un análisis de costoeficacia desde una perspectiva societaria que incluía la incertidumbre probabilística. Se midieron los resultados de los tratamientos tempranos basados en los resultados de un cultivo de tres meses y se modelaron resultados a largo plazo para facilitar la estimación del coste del programa integral de años de vida en función de la discapacidad (AVAD) evitada.

**Resultados:**

El programa integral costó 8.837 dólares estadounidenses (USD) por paciente tratado. Las bajas tasas de inscripción significaban que algunos de los costes fijos eran mayores por paciente que lo esperado. A pesar de que el programa integral parecía 30 veces más costoso que el anterior, tuvo como resultado unos mejores beneficios para la salud. El programa integral, que costó 639 USD (intervalo de credibilidad del 95%: de 112 a 1.322) por AVAD evitado, satisfizo el criterio de la Organización Mundial de la Salud en cuanto a intervenciones muy costoefectivas.

**Conclusión:**

El programa integral, que incluía un examen rápido, cuidados estándar y protección financiera, mejoró los resultados individuales de la tuberculosis multirresistente (MR) de una manera costoefectiva. Para apoyar los objetivos de salud globales posteriores a 2015, el programa integral debería expandirse a no residentes y otras áreas de China.

## ملخص

**الغرض:**

استقصاء مدى فعالية تكلفة البرنامج الشامل للتصدي لمرض السل المقاوم للأدوية الذي بدأ العمل به في أربعة مواقع في الصين في عام 2011.

**الطريقة:**

راجعنا سجلات 172 مريضًا يعانون من السل المقاوم للأدوية ممن تم تسجيلهم في البرنامج الشامل، وجمعنا البيانات الإدارية ذات الصلة من المستشفيات ووكالة الصحة العامة بالصين، وذلك في الفترة من 2011 إلى 2012. وفحصنا مجموعة تتكون من 81 مريضًا ممن تلقوا العلاج من السل المقاوم للأدوية في الفترة بين 2006 و2009 بغرض المقارنة. وأجرينا تحليلًا لمدى فعالية التكلفة من الناحية المجتمعية، حيث انطوى التحليل على احتمالات عدم التيقن من النتائج. وأجرينا قياسًا للنتائج الأولية للعلاج اعتمادًا على نتائج عملية المستنبتات التي استمرت لمدة 3 أشهر، واستخدمنا النتائج التي ظهرت على مدى أطول كنموذج لتيسير تقدير تكلفة البرنامج الشامل باستخدام سنوات العمر المصححة باحتساب مدد العجز (DALY) التي تم تفاديها.

**النتائج:**

بلغت تكلفة البرنامج الشامل 8837 دولارًا أمريكيًا للمريض الواحد الذي تلقى العلاج. وكانت المعدلات المنخفضة للتسجيل بالبرنامج تعني ارتفاعًا في بعض التكاليف الثابتة لكل مريض عن الحد المتوقع. وبالرغم من أن تكلفة البرنامج الشامل بدت أكثر ارتفاعًا بثلاثين ضعفًا مقارنةً بما سبق، فإنه أثمر عن نتائج أكثر جدوى من الناحية الصحية. إن البرنامج الشامل الذي تكلف 639 دولارًا أمريكيًا (بنسبة ثقة مقدارها 95%‏‏: من 112 إلى 1322 حالة) باستخدام سنوات العمر المصححة باحتساب مدد العجز التي تم تفاديها، قد اتفق مع معايير منظمة الصحة العالمية باعتباره تدخلاً فعالًا من حيث التكلفة إلى حد كبير.

**الاستنتاج:**

إن البرنامج الشامل، الذي اشتمل على الفحص السريع، والرعاية الصحية المتوافقة مع المعايير، والحماية المالية قد حسَّن من مستوى نتائج علاج السل المقاوم للأدوية المتعددة على المستوى الفردي بأسلوب فعال من حيث التكلفة. ويجب توسيع نطاق البرنامج الشامل ليضم غير المقيمين بالدولة ومناطق أخرى في الصين، وذلك لدعم الأهداف العالمية المنشودة لما بعد 2015 في مجال الصحة.

## 摘要

**目的:**

旨在调查 2011 年在中国四个地点实施的耐药结核病综合方案的成本效益。

**方法:**

在 2011 至 2012 年，我们查阅了 172 位接受该综合方案的耐药结核病患者的记录，并且从各家医院和中国的公共卫生机构收集了相关管理性数据。为了进行对比，我们调查了一组曾在 2006 年至 2009 年接受过耐药结核病治疗的 81 位患者。我们从社会视角对成本效益进行分析，其中包括概率不定性。我们在为期三个月的培养结果的基础上评估了早期治疗结果，并且以更长期的结果建模，以便于估计该综合方案每个伤残调整生命年 (DALY) 挽救生命的成本。

**结果:**

接受该综合方案的每位患者的治疗花费为 8837 美元 (US$) 。低接受率意味着每位患者的一些固定费用比预期的更高。尽管该综合方案的费用比之前的高 30 倍，但是它有更高的健康效益。该综合方案的每个伤残调整生命年挽救生命花费 639 美元（95% 置信区间：112 至 1322），符合世界卫生组织规定的一项极具成本效益的干预措施的标准。

**结论:**

这套包含了快速筛查、标准化护理和财务保障的综合方案以具有成本效益的方式，改善了多药耐药结核病患者的个人治疗成果。为了实现 2015 年后的全球健康目标，该综合方案的普及范围应扩大至非本地居民和中国的其他地区。

## Резюме

**Цель:**

Изучить экономическую эффективность комплексной программы по борьбе с лекарственно-устойчивым туберкулезом, запущенной в четырех китайских городах в 2011 г.

**Методы:**

В 2011–2012 гг. мы изучили медицинские карточки 172 пациентов, страдавших лекарственно-устойчивой формой туберкулеза и принявших участие в комплексной программе, и собрали соответствующие административные данные из больниц и агентства общественного здравоохранения Китая. Для сравнения рассматривалась когорта из 81 пациента, прошедшего лечение от лекарственно-устойчивой формы туберкулеза в период 2006–2009 гг. Нами был проделан анализ экономической эффективности с точки зрения социологии, который включал вероятностную неопределенность. Нами были измерены ранние результаты лечения (по данным культур, высеянных после трех месяцев лечения) и смоделированы исходы в более длительной перспективе, что позволило оценить выраженные в расчете на год жизни затраты на комплексную программу по утраченному времени в результате инвалидности (DALY), потерю которого удалось предотвратить.

**Результаты:**

Стоимость комплексной программы составила 8837 долларов США из расчета на одного пациента. Малое число участников программы означало, что некоторые фиксированные затраты из расчета на пациента были выше, чем ожидалось. Хотя данная комплексная программа оказалась в 30 раз дороже, чем предыдущая, ее результаты с точки зрения пользы для здоровья пациентов оказались лучше. Комплексная программа стоимостью 639 долларов США (95% доверительный интервал, диапазон от 112 до 1322 долларов США) из расчета на предотвращенный DALY удовлетворяет критериям Всемирной организации здравоохранения для экономически эффективного вмешательства.

**Вывод:**

Комплексная программа, включавшая быстрое скрининговое обследование, стандартизированное лечение и финансовую защиту, улучшила результат лечения у больных с МЛУ-формой туберкулеза и показала высокую экономическую эффективность. Чтобы достичь глобальных целей в сфере здравоохранения на период после 2015 года, данную комплексную программу следует распространить на нерезидентов страны и на другие регионы Китая.

## Introduction

China has more than halved tuberculosis prevalence and mortality since 1990, from 215 cases and 19 deaths per 100 000 population to 108 cases and 4 deaths per 100 000 population in 2010.^1^ However, in 2012, China had an estimated 59 000 notifications of multidrug-resistant (MDR) tuberculosis – resistant to both isoniazid and rifampicin – but only 1906 patients enrolled in treatment for MDR tuberculosis.^2^ If China’s attempts to end tuberculosis by 2035^3^ are going to succeed, it is necessary that patients with MDR tuberculosis receive adequate treatment.

In 2011−2014, the Chinese Center for Disease Control and Prevention (CCDC) piloted a comprehensive programme for drug-resistant tuberculosis in four areas of China: Chongqing municipality, Henan province, the Inner Mongolia autonomous region and Jiangsu province.^4^ The comprehensive programme included the screening of all patients with sputum-smear-positive pulmonary tuberculosis, rapid diagnosis, standardized care and financial protection against catastrophic health expenditure. In the previous baseline programme in effect in the four study areas, conventional drug-susceptibility testing was recommended for all newly-diagnosed patients with smear-positive pulmonary tuberculosis and for all previously-treated patients. In practice, such testing was confined to previously-treated patients and new patients who could afford to cover the full costs of such testing. The non-standard treatment lasted only as long as patients were willing and able to pay for it.

The aims of the study were to determine whether the comprehensive programme appeared cost-effective when compared against the baseline programme and a hypothetical no treatment, zero-cost alternative that we called the null programme.

## Methods

### Study perspective

We assessed cost–effectiveness from a societal perspective and therefore included direct medical costs incurred by hospitals, insurance schemes, patients and the Chinese CDC. We excluded the costs incurred by patients while seeking diagnosis or treatment from other providers before diagnosis under the comprehensive programme. We also excluded direct non-medical costs and any time costs incurred by patients and their caregivers. We had no reliable data on the excluded costs for the baseline programme, and the assessment and quantification of time costs are controversial. Using the World Health Organization’s cost–effectiveness threshold, we considered any intervention that cost less than China’s gross domestic product (GDP) per capita to avert a disability-adjusted life year (DALY) to be very cost-effective.^5^

### Comprehensive programme

In the comprehensive programme, sputum samples were collected from all patients who presented with sputum-smear-positive pulmonary tuberculosis either at a hospital or a dispensary maintained by the Chinese CDC. These samples were sent to a hospital laboratory and tested for *Mycobacterium tuberculosis* and resistance to rifampicin, using the rapid diagnostic Genechip test (CapitalBio, Beijing, China). All patients found positive for rifampicin-resistant *M. tuberculosis* were considered to be eligible for enrolment in the study if they were resident in one of the four study areas and had no contraindications. Although sputum samples were also cultured and then subjected to conventional drug susceptibility testing, enrolled patients were started on treatment for MDR tuberculosis before the results of such testing became available.

All enrollees were treated with the same standardized regimen. This regimen consisted of a six-month intensive phase – based on pyrazinamide, either amikacin or capreomycin, either levofloxacin or moxifloxacin, *p*-aminosalicylic acid and prothionamide – followed by an 18-month continuation phase, based on the same drugs except amikacin or capreomycin. A standard package included examinations before hospitalization, hospitalization up to two months, follow-up after discharge, health education and psychosocial support (available from the corresponding author).

To encourage collaboration in the referral, hospitalization and follow-up of patients, incentives were paid to the Chinese CDC and hospital staff and village doctors who were involved in the testing of the comprehensive programme (Table 1). A small transportation subsidy was paid to patients: about 15 United States dollars (US$) per hospital visit, for up to 13 visits.

**Table 1:** Incentives for hospital staff and village doctors involved in a comprehensive programme to treat drug-resistant tuberculosis, China, 2011–2012

Recipient	Type	Rationale	Payment
Hospital health workers in MDR-tuberculosis ward	Fixed recurring	Risk premium	US$ 8 per month
Hospital accountants	Fixed recurring	Data management	US$ 46 per month
Hospital health workers	Variable	Patient counselling	US$ 23 per enrolment
Other hospital workers	Fixed recurring	Increased workload	US$ 8 per month
City CCDC public health workers	Fixed recurring	Increased workload	US$ 8 per month
City CCDC laboratory workers	Fixed recurring	Increased workload	US$ 8 per month
City CCDC laboratories	Fixed recurring	Increased workload	US$ 231 per month
County CCDC public health workers	Fixed recurring	Increased workload	US$ 8 per month
County CCDCs	Variable	Referrals for diagnosis	US$ 2 per referral
County CCDCs	Variable	Successful enrolments	US$ 15 per enrolment
Village doctors	Variable	Injections	US$ 69 per month for up to 4 months per patient
Village doctors	Variable	Directly observed therapy	US$ 15 per month for up to 22 months per patient
Village doctors	Variable	Case management	US$ 8 per month for up to 22 months per patient

CCDC: Chinese Center for Disease Control and Prevention; MDR: multidrug-resistant; US$: United States dollars.

The comprehensive programme’s financial protection has been described in detail.^4^ In brief, the programme involved a move away from fee-for-service payment towards a standard package with controls on out-of-package fees and, by design, 90% of the fees within the standard package were reimbursed by insurance schemes and the programme. Under the baseline programme, reimbursement by the most common scheme, the New Rural Cooperative Medical Scheme, was 20−80% for inpatient services and 0−80% for outpatient services.

### Data collection

The data for the baseline programme came from a review of medical records and a previous survey. The data consisted of all people with confirmed MDR tuberculosis who were hospitalized for treatment in Henan province, the Inner Mongolia autonomous region or Jiangsu province between January 2006 and June 2009.^4^ Chongqing municipality was not included in the baseline study because, in the relevant period, it offered no culture confirmation of drug resistance.

Our investigation of the comprehensive programme was based on a detailed review of medical records and a survey of 73 enrollees – conducted after the enrollees had completed six months of treatment. We also included routinely collected data – mostly sputum-smear microscopy and culture results – for enrollees who gave their informed consent. The last routine data were collected in September 2012, after all the enrollees had completed at least six months of treatment. Costs for the full period of treatment were estimated according to the use of services and items defined in the standard package. Unit costs for outpatient clinic visits, sputum-smear microscopy, radiographs and hospital inpatient stays – i.e. bed days – were estimated, in each study area, using standard methods and templates.^6^ Unit costs for Genechip screening and conventional drug susceptibility testing were previously determined for each study area.^7^ We based our estimates of the unit costs of culture on our estimates of the unit costs of smear microscopy, multiplied by the ratio of the cost of culture to smear microscopy reported for six other sites in China (ratio range: 2.7–15).^8^

The cost of a bed day was multiplied by the mean number of bed days recorded for enrollees who had been discharged from hospital by September 2012 and then by the total number of enrollees. The unit cost of a Genechip test was multiplied by the total number of patients screened. For conventional drug susceptibility testing, outpatient visits, smear microscopy, culture and chest X-rays, unit costs were multiplied by quantities indicated in the standard care package and by the number of enrollees. The total cost of the drugs used within the standard package was extracted from hospital fee schedules. For all other tests and drugs used within or outside the standard package, we extracted hospital charges from the relevant patient records. We reduced the cost of drugs by 15% to remove the hospitals’ permitted mark-up.

Administrative data were collected from the facilities maintained by the Chinese CDC, at both city and county levels. Project and data management costs were estimated by multiplying the number of full-time equivalent staff times their gross wages and adding the cost of meetings between the Chinese CDC and the hospitals involved in the comprehensive programme. Health promotion costs were estimated from the cost of the relevant printed materials and visits of staff from city-level facilities to county-level facilities. Infection control costs at county level included the costs of renovating sputum collection rooms and purchasing surgical masks and N95 respirators. The cost of testing a sputum sample in a hospital was calculated from the cost of each sputum sample collected, registered and transported to the hospital for screening times, the number of such samples.

Administration costs involving the Chinese CDC were based on data collected over a year and therefore had to be extrapolated to cover the 24 months of the standard treatment regimen. The costs of project and data management, incentives, infection control, health promotion and training were divided into two categories: variable costs and fixed costs. Variable cost items were multiplied by the total number of treatment days and then divided by the number of days of treatment elapsed. Fixed costs were classified as recurring annual costs or non-recurring, one-time costs. Non-recurring fixed costs for assets with a useful life of more than one year were annualized – assuming that office and laboratory equipment would remain useful for three to five years and that buildings would remain useful for 20 years. We used a discount rate of 3% per year for all costs.

In a probabilistic sensitivity analysis, we allowed total cost to vary by an additional 20% above and below the levels of uncertainty embedded in the unit costs.

We inflated the baseline costs to 2011 prices using a price deflator based on Chinese GDP.^9^ We reflected sampling uncertainty in a gamma distribution. Although all costs were initially recorded in Chinese yuan (¥), we report them in US$ for the year 2011, when US$ 1.00 had a mean value of ¥ 6.5.

### Modelling and analysis

We constructed mathematical models using R software (R foundation, Vienna, Austria).^10^^,^^11^ The mc2d package allowed for probabilistic sensitivity analysis of the model parameters along two dimensions.^12^ To the *y* dimension we attached parameter uncertainty – i.e. uncertainty around the final two-year costs and outcomes – and to the *x* dimension we attached all other – i.e. model and parameter – uncertainty. This two-dimensionality allowed us to assess how uncertainty about final costs and outcomes affected the results and to estimate the expected value of so-called partially perfect information. We simulated 500 values along each dimension. We tested for robustness of the results to 10 000 simulations along a single dimension.

We predicted two-year treatment outcomes from the results of culturing sputum samples collected three months after the initiation of treatment. Using a systematic review, we assumed that for negative month three test results, the probability of successful treatment (cure or completion of treatment) is 75%, death is 9%, treatment default is 13% and treatment failure is 3%.^13^ We used *β* distributions for the outcome classes to generate multinomially distributed random number vectors representing the number of patients who were culture-negative at three months and still on treatment at six months. We also generated random number vectors for patients who were culture-positive at three months and still on treatment at six months. We summed these vectors with the known number of deaths and defaulters at six months to obtain the final distributions for the numbers of successes, deaths, defaults and failures at two years.

The decision trees for the baseline and null programmes are available from the corresponding author, as well as descriptions of the long-term mortality parameters – e.g. the probability of relapse and subsequent death of a patient who had been successfully treated – and transmission parameters. Modelling of transmission was based on the duration of the patients’ infectious period and decision trees and parameters that were consistent with those used in earlier cost–effectiveness analyses of interventions against MDR tuberculosis.^14^^–^^16^ We assumed that secondary patients – patients generated by the primary patients – would have the same costs and effects as the primary – i.e. index – cases.

### Ethical approval

This economic evaluation is based in part on secondary data published as a result of a pilot study that was reviewed and approved by the Tuberculosis Operational Research Ethics Review Committee of the Chinese Ministry of Health. The collection of the other data that we used satisfied the WHO Ethics Review Committee’s conditions for exemption.

## Results

### Detection and enrolment

Although all 2816 notified smear-positive patients in the four study areas in 2011−2014 should have been screened for rifampicin resistance, data on such screening were only available for 2244 (80%) of them. Overall, 243 (11%) patients tested positive for resistance to rifampicin and 172 of these were enrolled. Subsequent conventional drug susceptibility testing revealed that 112 (65%) of the enrolees carried *M. tuberculosis* that was resistant to both rifampicin and isoniazid. There were several reasons for non-enrolment, including the presence of clinical complications.^4^

In the baseline programme, only 92 patients with MDR tuberculosis were identified over a period of 2.8 years and only 81 (88%) of those patients were started on treatment for MDR tuberculosis.

The characteristics of the enrollees are summarized in Table 2. The proportion of enrollees insured under the New Rural Cooperative Medical Scheme exceeded the proportion of MDR tuberculosis patients detected in the baseline programme who were insured under the same scheme. To adjust for this difference in insurance coverage, we weighted the costs so that the estimated costs for the baseline programme were reduced.

**Table 2:** Characteristics of patients eligible for a comprehensive programme to treat drug-resistant tuberculosis, China, 2011–2012

Characteristic	No. (%)		*P*^a^	No. (%)	*P*^b^
	Eligible (*n* = 243)	Enrolled (*n* = 172)		Surveyed (*n* = 73)	
**Age, years**					
15–24	26 (11)	22 (13)	0.22	8 (11)	0.25
25–34	31 (13)	21 (12)		8 (11)	
35–44	46 (19)	30 (17)		12 (16)	
45–54	62 (26)	49 (28)		22 (30)	
≥ 54	77 (32)	50 (29)		23 (32)	
Unknown	1 (0.4)	0 (0)		0 (0)	
**Sex**					
Male	176 (72)	128 (74)	0.17	57 (78)	0.86
Female	67 (28)	44 (26)		16 (22)	
**Insurance**					
Urban workers	18 (7)	12 (7)	0.44	6 (8)	0.34
Urban residents	23 (9)	19 (11)		7 (10)	
Rural	135 (56)	110 (64)		52 (71)	
Other	6 (2)	6 (3)		3 (4)	
None	27 (11)	25 (15)		5 (7)	
Unknown	34 (14)	0 (0)		0 (0)	
**Employment status**					
Employed	45 (19)	28 (16)	0.00	9 (12)	0.01
Unemployed	170 (70)	143 (83)		63 (86)	
Unknown	28 (12)	1 (0.6)		1 (1)	
**Household income per capita, yuan**^c^					
0 to 2300	17 (7)	15 (9)	0.15	6 (8)	0.49
2300 to 5000	78 (32)	66 (38)		28 (38)	
5000 to 10 000	35 (14)	31 (18)		14 (19)	
> 10 000	74 (30)	57 (33)		22 (30)	
Unknown	39 (16)	3 (2)		3 (4)	
**HIV status**^d^					
Negative	243 (100)	172 (100)	0.90	73 (100)	0.90
Positive	0 (0)	0 (0)		0 (0)	
**Patient category**					
New	67 (28)	37 (22)	0.24	11 (15)	0.52
Relapse	69 (28)	53 (31)		21 (29)	
Initial treatment failure	16 (7)	11 (6)		5 (7)	
Retreatment failure/chronic case	77 (32)	59 (34)		31 (42)	
Other	14 (6)	12 (7)		5 (7)	

HIV: human immunodeficiency virus.

^a^ For enrolled versus not enrolled, from *χ^2^* tests – or *t*-tests for the continuous age and income variables – with clustering based on the four study areas.

^b^ For surveyed versus not surveyed, from *χ^2^* tests – or *t*-tests for the continuous age and income variables – with clustering based on the four study areas.

^c^ For the 12 months before the patient’s diagnosis.

^d^ Self-reported.

Notes: Inconsistencies arise in some values due to rounding. The conversion rate was 1.0 United States dollars to 6.5 Chinese yuan for 2011.

### Costs

**Fig. 1:**
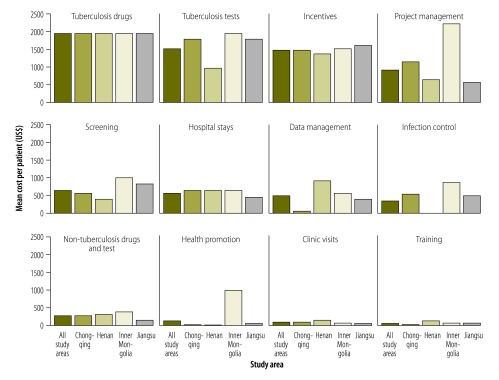
Mean costs per patient in a comprehensive programme to treat drug-resistant tuberculosis, China, 2011–2012

The total cost of the comprehensive programme was US$ 8837 (95% credible interval, CrI: 6649 to 10 962) per patient. Drugs and tests within the standard package made up 23% (US$ 1961) and 17% (US$ 1532) of the total, respectively (Fig. 1). In the baseline programme, medical fees totalled US$ 1235 (95% CrI: 1149 to 1329) per patient. Basic treatment costs were lower in the baseline programme than in the comprehensive programme – in part because so few patients in the baseline programme completed treatment.

US$: United States dollars.

Notes: All costs have been adjusted to 2011 prices.

In the comprehensive programme, US$ 1506 per patient – i.e. 17% of the total – was spent on incentives and enablers, including variable incentives to village doctors for community-based outpatient treatment, a mix of fixed and variable incentives to hospital workers, variable incentives to the Chinese CDC’s county workers, transportation subsidies to patients and fixed incentives to the Chinese CDC’s city workers.

In the comprehensive programme, project management costs were considerably higher in the Inner Mongolia autonomous region – where fixed costs were spread over only 17 patients – than in the other three study areas. The same region also faced relatively high transportation costs and these contributed to relatively high screening and health promotion costs. In the first 12 months of the comprehensive programme, bed occupancy ranged from 33% to 57% – i.e. from the six beds in Inner Mongolia autonomous region occupied for a total of 723 bed days to the 12 beds in Henan province occupied for a total of 2496 bed days. The cost of hospital stays totalled US$ 585 per patient and US$ 292 per patient were spent on drugs and tests outside the standard package. The total cost of screening 2244 smear-positive patients, then diagnosing and treating 172 patients for MDR tuberculosis, was estimated at US$ 1.5 million or about 30 times the total cost of the baseline programme. When we included the costs of treatment of all future secondary patients generated by the primary patients, the total estimated cost of the comprehensive programme became US$ 4.2 million.

### Effectiveness

**Fig. 2:**
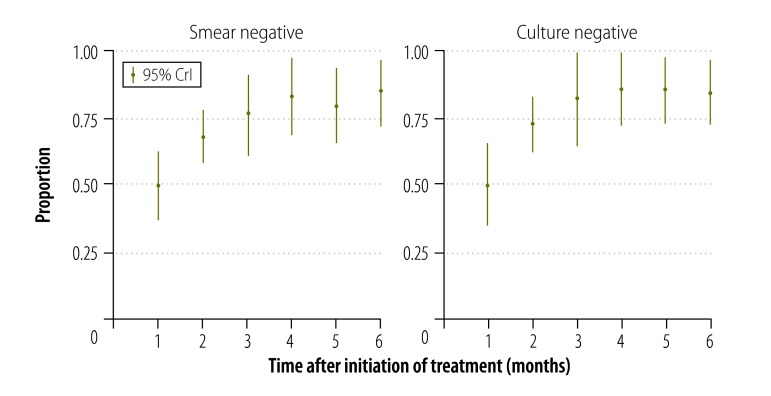
Patients with a negative *Mycobacterium tuberculosis* test, China, 2011–2012

In the comprehensive programme, 4% (7) of the enrollees died and 15% (26) defaulted within the first six months. For the 139 enrolees who remained on treatment for six months, 83% (115) had negative sputum cultures by their third month of treatment (Fig. 2). There was no significant increase in negative cultures between the third and sixth months. We did not find a statistically significant difference in outcomes between study areas or patient categories.

CrI: credible interval.

Notes: The sample includes all 139 enrollees of rifampicin-resistant or multidrug-resistant tuberculosis who were enrolled in the comprehensive programme and remained on treatment six months after initiating treatment. The CrIs reflect sampling uncertainty as well as uncertainty relating to missing data.

In the baseline programme, two (2%) of the 81 patients who started on treatment were still on treatment after six months. The other 66 died or defaulted; six (9%) died within six months of starting treatment.

The predicted two-year success rate for the patients enrolled in the comprehensive programme was 0.563 (Table 3). The corresponding value for the enrollees who showed resistance to both rifampicin and isoniazid was similar. The success rates predicted for the comprehensive programme were much higher than the corresponding rates for the baseline programme, but similar to those reported in some relatively high-income settings in China.^17^^,^^18^

**Table 3:** Predicted treatment outcomes two years after initiating treatment for drug-resistant tuberculosis, China

Outcome	Best estimate of probability (95% CrI)				
	Comprehensive programme			Baseline programme	Beijing and Shanghai^b^
	Rifampicin-resistant (*n* = 172)	MDR tuberculosis^a^ (*n* = 112)		MDR tuberculosis (*n* = 81)	MDR tuberculosis (*n* = 807)
Success^c^	0.563 (0.488 to 0.628)	0.562 (0.473 to 0.643)		0.023 (0.000 to 0.029)	0.538 (0.504 to 0.572)
Death	0.127 (0.087 to 0.172)	0.136 (0.089 to 0.188)		0.091 (0.088 to 0.103)	0.026 (0.017 to 0.038)
Default	0.261 (0.215 to 0.314)	0.244 (0.188 to 0.304)		0.886 (0.882 to 0.912)	0.131 (0.054 to 0.229)
Failure	0.041 (0.012 to 0.087)	0.050 (0.009 to 0.098)		0.001 (0.000 to 0.015)	0.304 (0.198 to 0.410)

CrI: credible interval; MDR: multidrug-resistant.

^a^ Included 10 patients with extensively drug-resistant tuberculosis.

^b^ Treatment outcomes obtained from a meta-analysis using random effects of results for MDR-TB patients.^17^^,^^18^

^c^ Success outcome was defined as either cure or completion of treatment.

When considering only the primary patients that were enrolled, we estimated that the comprehensive programme would avert over their lifetimes an estimated 41 tuberculosis (TB) deaths compared with the baseline programme and 52 TB deaths compared with the null programme (Table 4). Suboptimal enrolment in the comprehensive programme presumably led to some avoidable deaths.

**Table 4:** Estimated costs, effects and cost–effectiveness of the comprehensive, baseline and null programmes to treat drug-resistant tuberculosis, China, 2011–2012

Variable	Best estimate for programme (95% CrI)		
	Comprehensive	Baseline	Null
**Rifampicin-resistant patients**			
No. among notified tuberculosis patients	305 (300 to 310)	305 (300 to 310)	305 (300 to 310)
No. detected	243 (–)	36 (31 to 41)	0 (–)
No. detected and enrolled	172 (149 to 193)	30 (25 to 36)	0 (–)
**Costs (millions of US$)**^a^			
Direct medical costs			
Primary patients	1.57 (1.20 to 1.93)	0.04 (0.03 to 0.05)	0 (–)
Primary and secondary patients	4.12 (2.22 to 8.00)	0.02 (0.06 to 0.43)	0 (–)
Incremental costs relative to baseline			
Primary patients	1.52 (1.15 to 1.90)	–	–
Primary and secondary patients	3.95 (2.12 to 7.64)	–	–
Incremental costs relative to null			
Primary patients	1.57 (1.20 to 1.93)	0.04 (0.03 to 0.05)	–
Primary and secondary patients	4.12 (2.22 to 8.00)	0.02 (0.06 to 0.43)	–
**Effects**			
Long-term tuberculosis deaths			
Primary patients	119 (100 to 139)	160 (144 to 176)	171 (154 to 187)
Primary and secondary patients	356 (184 to 781)	1 074 (305 to 3648)	902 (330 to 3077)
Deaths averted relative to baseline			
Primary patients	41 (28 to 54)	–	–
Primary and secondary patients	717 (99 to 2994)	–	–
DALYs averted relative to baseline			
Primary patients	843 (551 to 1 191)	–	–
Primary and secondary patients	15 789 (1879 to 67 251)	–	–
Deaths averted relative to null			
Primary patients	52 (38 to 66)	11 (9 to 14)	–
Primary and secondary patients	546 (122 to 2438)	–171 (–670 to 61)	–
DALYs averted relative to null			
Primary patients	1 072 (749 to 1475)	229 (165 to 317)	–
Primary and secondary patients	11 598 (2342 to 54 451)	–4191 (–15 403 to 1113)	–
**Cost–effectiveness (US$)**^a^			
Cost per death averted relative to baseline			
Primary patients	38 258 (24 386 to 58 723)	–	–
Primary and secondary patients	14 257 (1885 to 30 130)	–	–
Cost per DALY averted relative to baseline			
Primary patients	1879 (1143 to 2948)	–	–
Primary and secondary patients	715 (84 to 1583)	–	–
Cost per death averted relative to null			
Primary patients	30 564 (20 449 to 43 973)	3536 (3082 to 4109)	–
Primary and secondary patients	12 772 (2500 to 25 442)	NA	–
Cost per DALY averted relative to null			
Primary patients	1502 (949 to 2251)	174 (130 to 229)	–
Primary and secondary patients	639 (112 to 1322)	NA	–

CrI: credible interval; DALY: disability-adjusted life-year; NA: not available; US$: United States dollars.

^a^ Adjusted to 2011 prices.

Note: Primary patients are patients presenting during the pilot; secondary patients are patients generated by the primary (index) patients and that will present sometime in the future, after infection and eventual activation of the disease.

When we extended our predictions to cover primary patients plus any secondary patients infected by primary patients, we found that the comprehensive programme would avert over their lifetimes an estimated 717 TB deaths and 15 800 DALYs compared with the baseline programme and 546 TB deaths and 11 600 DALYs compared with the null programme. There was some indication that, by extending the life of patients but not achieving a cure, the baseline programme might ultimately avert fewer DALYs than the null programme.

### Cost–effectiveness

When we included all enrollees in our calculations, we found that, per death averted, the comprehensive programme cost US$ 14 257 compared with the baseline programme and US$ 12 772 compared with the null programme (Table 4). The corresponding costs per DALY were US$ 715 and US$ 639, respectively.

**Fig. 3:**
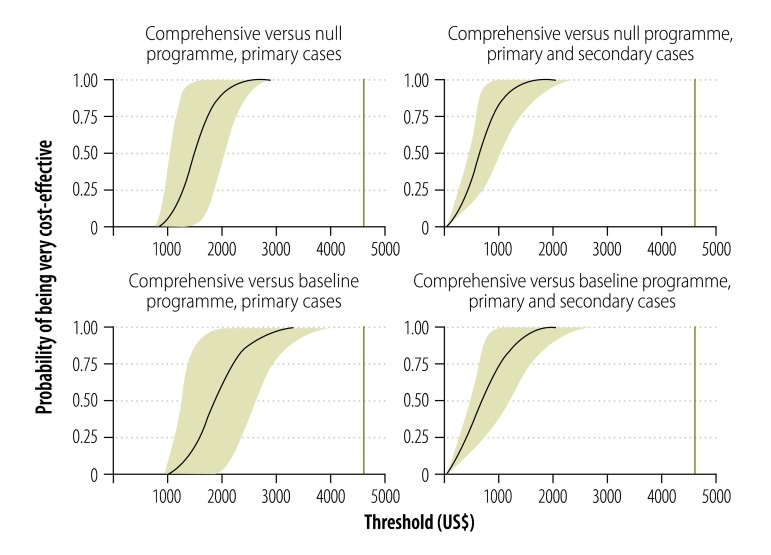
Probability of cost–effectiveness at different thresholds for a comprehensive programme to treat drug-resistant tuberculosis, China, 2011–2012

Fig. 3 shows the probability that the comprehensive programme will be very cost-effective at different thresholds of cost–effectiveness. We considered a conservative threshold to be US$ 4615 per DALY averted. This value is roughly equivalent to the GDP per capita in Henan province in 2011 and lower than the GDP per capita for the other three study areas and for mainland China as a whole.^19^ The probability that the comprehensive programme is very cost-effective – compared with either the baseline programme or the null – was found to be more than 99% under all of the scenarios that we considered.

US$: United States dollars.

Notes: The comprehensive programme was compared with baseline and null programmes. The comprehensive programme was considered very cost-effective if it cost less than China’s per capita gross domestic product (vertical green line) to avert a disability-adjusted life-year.^5^ In each plot, the grey-shaded area indicates the uncertainty around the final two-year costs and outcomes for the full cohort.

The uncertainty around the final outcomes had a negligible influence on our main findings (Fig. 3).

## Discussion

Our results add to the data on the cost and cost–effectiveness of treatment for MDR tuberculosis.^20^ Earlier studies looked at treatment provided free of charge to patients by public and non-profit providers.^14^^–^^16^ The present study included the costs associated with rapid screening and financial protection and considered the effects of such screening and protection on access and adherence. The costs of incentives and non-tuberculosis-specific drugs and tests in our study areas of China were higher than those reported in related studies in other countries – reflecting the unique and complex Chinese system of publicly-owned but profit-seeking hospitals.

The comprehensive programme appeared to offer a very cost-effective approach to the control of MDR tuberculosis in all four areas where it was piloted. Early outcomes for the enrollees were much improved and the financial protection from controls on out-of-package fees and higher rates of reimbursement were associated with much better treatment adherence. Aggregate outcomes for the comprehensive programme would have been better if more patients had been enrolled, but some patients may not be able to afford the standard package even with 90% reimbursement. Due to extensive internal migration, coverage of this programme should be expanded to migrants within the study areas and scaled up to cover China as a whole. Given China’s large share of the world’s drug-resistant tuberculosis burden, implementation of the comprehensive programme across China will help ensure that the world meets its tuberculosis-related targets for the post-2015 development agenda.

## Conclusion 

The comprehensive programme improved individual outcomes for MDR tuberculosis in a cost-effective manner. To support post-2015 global heath targets, the comprehensive programme should be expanded to non-residents and other areas of China. This programme could also be applicable to other countries with low rates of detection and poor treatment outcomes. However, China is a middle-income country with a low prevalence of human immunodeficiency virus (HIV). Further research will be required to assess the cost–effectiveness of this approach in low-income settings with a high prevalence of HIV.
